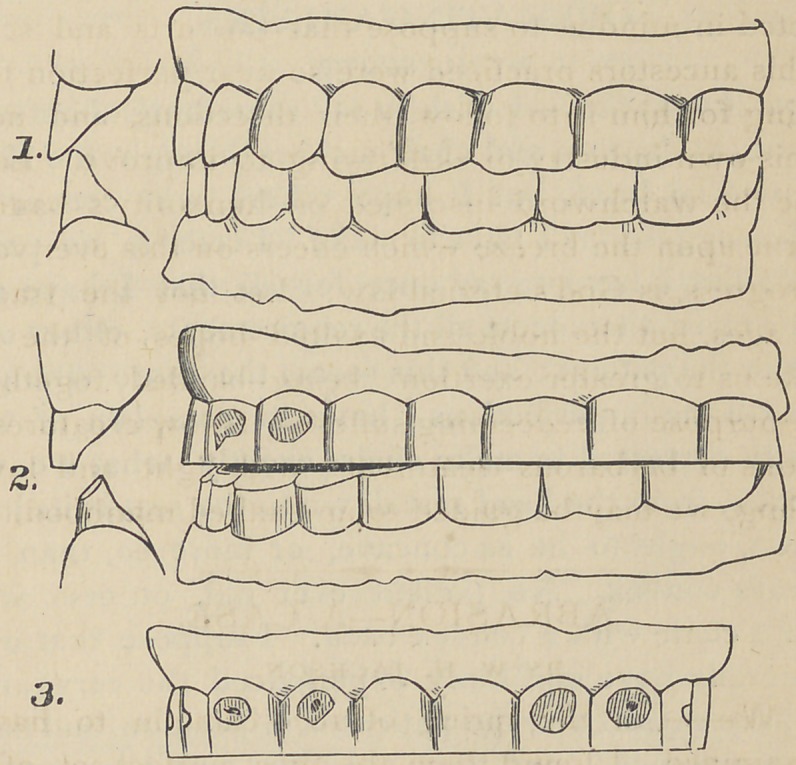# Abrasion—A Case

**Published:** 1873-06

**Authors:** W. H. Jackson


### ABRASION—A CASE.

BY W. H. JACKSON.

Mrs. W——, in the spring of 1870 came in to have her teeth examined. I found them the most perfect set of teeth I ever saw, each tooth occupying its proper position, well developed, cusps full and round, and to all appearances in the most perfect state of health. I remarked to her that I wished every one had as perfect teeth as she.

See Fig. 1 in the plate. The cusps and points of enamel are all perfect. The cutting edges of the incisors are sharp and sustain their normal relation to each other.

Last March, 1873 she called again. Upon examination I found the cutting and grinding surfaces of the superior teeth extensively abraded having the appearance of having been held upon a flat-stone and ground until the cusps were all gone, leaving no enamel upon the articulating surfaces. The enamel on labial surface of the superior central incisors is extremely thin, the left superior lateral incisor has lost three-fourths its enamel reaching to the cutting edge and the left superior cuspid one half, is much hollowed.; the left bicuspids, right laterial incisor, cuspid and bicuspids all have lost a small portion of the enamel on the labial surface, and it is only upon close observation that the dividing line between the enamel and dentine is discovered.

Fig. 2. represents the teeth as abraded during the period of two years ending March, 1873.

The incisors, cuspids and first bicuspids are beveled at an angle of 45° from the lingual surface downwards and outwards, all being beveled alike with smooth polished surfaces, the inferior second bicuspids and molars have smooth flat articulating surface like their superior antagonists. It is impossible to bring the front-teeth in contact, as far back as the bicuspids, hence it is impossible to account for it by mechanical abasion. Patient's age, 38, married, no children, lymphatic temperament, has enjoyed and is now in good health, gums light pink, hard, no tendency to sponginess, no dyspepsia, bowels regular. Teeth a little more than medium white, with yellowish tinge, enamel of medium density, dentine the same, have not tested for acid in secretions, but shall at first opportunity. Would like to hear from others as to treatment in such a case.

## Figures and Tables

**Figure f1:**